# Multivariate Feature Selection of Image Descriptors Data for Breast Cancer with Computer-Assisted Diagnosis

**DOI:** 10.3390/diagnostics7010009

**Published:** 2017-02-14

**Authors:** Carlos E. Galván-Tejada, Laura A. Zanella-Calzada, Jorge I. Galván-Tejada, José M. Celaya-Padilla, Hamurabi Gamboa-Rosales, Idalia Garza-Veloz, Margarita L. Martinez-Fierro

**Affiliations:** 1 Unidad Académica de Ingeniería Eléctrica, Universidad Autónoma de Zacatecas, Jardín Juarez 147, Centro, 98000 Zacatecas, Zac, Mexico; ericgalvan@uaz.edu.mx (C.E.G.-T.); gatejo@uaz.edu.mx (J.I.G.-T.); hamurabigr@uaz.edu.mx (H.G.-R.); 2 Facultad de Ciencias, Universidad Autónoma de San Luis Potosí, Lateral Av. Salvador Nava s/n., 78290 San Luis Potosí, SLP, Mexico; lau_zanella@hotmail.com; 3 CONACYT – Universidad Autónoma de Zacatecas – Jardín Juarez 147, Centro, 98000 Zacatecas, Zac, Mexico; 4 Unidad Académica de Medicina Humana y Ciencias de la Salud, Universidad Autónoma de Zacatecas, Jardín Juarez 147, Centro, 98000 Zacatecas, Zac, Mexico; idaliagv@uaz.edu.mx (I.G.-V.); margaritamf@uaz.edu.mx (M.L.M.-F.)

**Keywords:** breast cancer, mammography image features, mammography descriptors, CAD, multivariate model, genetic algorithm, machine learning algorithms

## Abstract

Breast cancer is an important global health problem, and the most common type of cancer among women. Late diagnosis significantly decreases the survival rate of the patient; however, using mammography for early detection has been demonstrated to be a very important tool increasing the survival rate. The purpose of this paper is to obtain a multivariate model to classify benign and malignant tumor lesions using a computer-assisted diagnosis with a genetic algorithm in training and test datasets from mammography image features. A multivariate search was conducted to obtain predictive models with different approaches, in order to compare and validate results. The multivariate models were constructed using: Random Forest, Nearest centroid, and K-Nearest Neighbor (K-NN) strategies as cost function in a genetic algorithm applied to the features in the BCDR public databases. Results suggest that the two texture descriptor features obtained in the multivariate model have a similar or better prediction capability to classify the data outcome compared with the multivariate model composed of all the features, according to their fitness value. This model can help to reduce the workload of radiologists and present a second opinion in the classification of tumor lesions.

## 1. Introduction

Breast cancer is an important public health problem worldwide, and it is the most common type of cancer among women. It continues to increase, especially in developing countries [1]. According to the World Health Organization (WHO), approximately 508,000 women died of breast cancer in 2011 [2], and according to the Instituto Nacional de Estadística y Geografía (INEGI), in 2004, 13.3% of women deaths were associated with breast cancer in Mexico, making breast cancer the second cause of death in women [3]. According to the National Cancer Institute of Canada, one out of 8.9 is the probability that women will develop this disease during their lifetime, and the probability of death is one out of 26.8 [4].

One of the biggest problems associated with this disease is the late detection, which is an important fact reducing the survival rates; localized cancer leads to a 5-year survival rate of 97.5%, whereas cancer that has spread to distant organs has a 5-year survival rate of only 20.4% [5]. Another important fact of breast cancer is that is impossible to prevent (excepting prophylactic mastectomy), because its causes remain unknown [6,7].

A set of diagnosis methods for breast cancer have been studied by experts, looking for early detection, finding that the primary method—breast self-examination—is not adequate, since there is no evidence of a reduction in the mortality rate due to this disease in women who practice breast self-examination against to those who do not [8,9,10].

Nowadays, there are several techniques that use a computational approach to estimate relationships between several elements and breast cancer [11]; however, a well-known tool for early detection of breast cancer is mammography screening [12,13], which has proved being effective in reducing breast cancer mortality rates by 30%–70% [14]. The problem with mammograms is that they can be difficult to interpret because the sensitivity can be affected by the image quality and the radiologistś level of expertise [15].

To solve this problem, recent studies have implemented algorithms to improve the detection of breast cancer and to provide assistance to radiologists by computer-assisted diagnosis (CAD), aiming to obtain a better prognosis for the patient, and helping the radiologists to detect abnormalities earlier than if traditional procedures were used [16]. CAD can improve the quality of the image and give a second reading of screening mammograms [17]. Additionally, CAD algorithms can estimate the probability that a cancer lesion is malignant or benign [18]. It is known that benign tumors cannot spread or invade other parts of the body, while malignant tumors grow rapidly and may invade surrounding tissues, causing damage [19,20]. These algorithms normally use different descriptive cancer features, but a standard set of features that gives an accurate classification has not been found [21]. Toward this aim, several works have been proposed. For instance, Patel et al. proposed a new method for breast image segmentation for early detection of breast cancer based on the detection of micro-calcification and a computer-based decision system using an adaptive k-means clustering algorithm [22]. A new different CAD scheme focusing on clinical applications was proposed by Doi Kunio et al. [23], proposing a methodology to distinguish—in similar radiological images—the malignant lesions from benign lesions, thus achieving a diagnosis of breast cancer. Interesting approaches have been put forth that make use of machine learning techniques; for instance, R. Ramani et al. [24] proposed a new approach to classify masses in the mammography image using a feature extraction approach. Features extracted from mammography achieved by using Symlet, SVD, and weighted histogram were used to train a classification model (naïve Bayes, random forest, and neural network algorithms) and classify tumors as benign or malignant given their features. Anther proposal using machine learning algorithms is presented by J. Dheeba et al. [25], they proposed a CAD system to detect breast cancer in mammograms through optimized swarm intelligence by neural networks that detect cancer. The primary focus is the neural network optimization to improve the detection accuracy, proving that this method has a better behavior than the others, and actually works. Finally, an interesting proposal using a digital image processing approach merged with a feature extraction approach is presented by Karahaliou et al. [26], proposing a wavelet-based contrast enhancement method to find the texture descriptors extracted from mammograms which can contribute to cancer diagnosis. To validate the texture features’ ability to discriminate malignant from benign lesions, a k-Nearest Neighbor (KNN) classifier was used. Finally, to know the performance, the area under the curve (AUC) and the receiver operating characteristic (ROC) were considered, showing promising results in CAD of breast cancer that may contribute to the reduction of unnecessary biopsies.

However, in all of the approaches presented above, the importance of features to describe the behavior of the tumors is absent, leading to each author proposing different features for extraction; therefore, the contribution of this paper is the search for a multivariate classification model, aiming to a CADx system based on: mammography assessments, ultrasound image features, clinical history, lesion segmentation, and selected pre-computed image-based descriptors, analyzing the behavior of each feature and the impact on the accuracy of the classification using a genetic algorithm approach.

The purpose of this work is to classify between malignant and benign tumors using a specific set of features. Results suggest that the multivariate model with the texture descriptor features obtained has higher prediction capabilities. Therefore, using this multivariate model can be very useful for radiologists to have a second opinion and improve the accuracy of their classification predictions.

This paper is organized as follows. The background for this study is described in Section 2. Data set description and methodology used to study the impact of features to classify tumors are presented in Section 3. In Section 4, we present the experiments and results. Finally, a discussion of results and conclusions drawn from them are presented in Section 5.

## 2. Background

In this section, the equations used to describe breast features and machine learning algorithms used as cost function throughout this study are presented.

### 2.1. Breast Image Descriptions

The features evaluated by the classification methods are a set of equations that describe different properties of the mammograms. Said properties can be grouped into intensity, texture, shape, and location descriptors.

#### 2.1.1. Intensity Descriptors Computed from the Grey-Levels of the Pixels Inside the Lesion’s Contour Identified by the Radiologists

(1) $$i\_skewness=\frac{\frac{1}{n}\Sigma_{i}{\left(X_{i}-\bar{X}\right)}^{3}}{{\left(\sqrt{\frac{1}{n}\Sigma_{i}{\left(X_{i}-\bar{X}\right)}^{2}}\right)}^{3}}$$

Equation (1) calculates the skewness of the segmented lesion, with *n* being the number of pixels inside the region delimited by the contour, $X_{i}$ being the grey level intensity of the $i^{th}$ pixel inside the contour, and $\bar{X}$ the $X_{i}$s mean.

#### 2.1.2. Texture Descriptors Computed from the Grey-Level Co-Occurrence Matrix Related to the Bounding Box of Lesion’s Contour Identified by the Radiologists

(2) $$t\_homo=\Sigma_{i}\Sigma_{j}\frac{1}{1+{\left(i-j\right)}^{2}}p\left(i,j\right)$$

Equation (2) calculates the homogeneity of the segmented lesion, with *i* and *j* being the number of grey-levels, *p* being the grey-level co-occurrence matrix, and thus, p(i,j) is the probability of pixels with grey-level *i* occurring together with pixels with grey-level *j*.

(3) $$t\_inf2h=\sqrt{1-exp(2\left(\Sigma_{i}\Sigma_{j}P_{x}\left(i\right)P_{y}\left(j\right)log\left(P_{x}\left(i\right)P_{y}\left(j\right)\right)-\Sigma_{i}\Sigma_{j}p\left(i,j\right)log\left(p\left(i,j\right)\right)\right))}$$

Equation (3) calculates the information measure of correlation, with $P_{x}$ and $P_{y}$ being the partial probability density functions.

(4) $$t\_corr=\frac{\Sigma_{i}\Sigma_{j}\left(ij\right)p\left(i,j\right)-\mu_{x}\mu_{y}}{\sigma_{x}\sigma_{y}}$$

Equation (4) calculates the correlation of the segmented lesion, with $\mu_{x}$, $\mu_{y}$, $\sigma_{x}$, and $\sigma_{y}$ being the means and standard deviations of $P_{x}$ and $P_{y}$, the partial probability density functions.

(5) $$t\_denth=-\Sigma_{i}P_{x-y}\left(i\right)log\left(P_{x-y}\left(i\right)\right)$$

Equation (5) calculates the entropy difference of the segmented lesion, with $P_{x-y}$ being the probability of the co-occurrence matrix coordinates subtracting i = |x-y|.

#### 2.1.3. Shape and Location Descriptors of the Lesion’s Contour Identified by the Radiologists

(6) s_perimeter=length(E)

Equation (6) is used to calculate the perimeter of the segmented lesion, with *E* being the edge pixels that belong to the segmented lesion.

(7) $$s\_circularity=4\pi \frac{area}{perimeter^{2}}$$

Equation (7) is used to calculate the circularity of the segmented lesion, with the area and the perimeter of the segmented lesion

(8) $$s\_elongation=\frac{m}{M}$$

Equation (8) calculates the elongation of the segmented lesion, with *m* being the minor axis and *M* the major axis of the ellipse that has the same normalized second central moments as the region surrounded by the contour.

(9) s_x_center_mass=normalized coordinates of the center of mass of O

Equation (9) calculates the center of mass of the segmented lesion, with *O* being the edge of the pixels.

### 2.2. Classification Methods

The methods evaluated in this research consist of three different classification algorithms: (i) Random Forest (RF), (ii) Nearest Centroid (NC), and (iii) K Nearest-Neighbors (K-NN). These approaches were considered for this work in order to evaluate the performance of methods that belong to different categories. RF is a nonlinear supervised sparse regression–based method [27], while K-NN is a supervised instance-based method [28]. On the other hand, the NC method can be considered a hybrid approach that encompasses an instance-based approach as well as a statistical one [28].

#### 2.2.1. Random Forest

RF is a classification method proposed by Breiman et al. [29]. It is a robust machine learning technique that can handle classification problems based on bagging and random feature selection; this technique has been widely used in different medical areas, such as brain tissue segmentation [30].

This algorithm consists of a set of decision trees based on split nodes and leaf nodes, where each tree is composed of randomly selected features. These trees grow on their split nodes, depending on the values of their random feature vector, evaluating each of these values and the incoming samples (provided with the same distribution), and depending on the features evaluation, pass it to the left or right offspring. The leaf nodes store the statistic of the sample that arrives.

The algorithm process consists of two stages: training and testing. In the training stage, multiple decision trees are constructed with the entire set of features. The trees are initially generated evaluating the complete data; then, in the ith tree (internally selected by the algorithm depending on the number of subjects), the algorithm selects a subset of data for training which is randomly sampled with a replacement from the entire data set; then, the subset of data selected recursively train each node in the tree, starting from the root node or the top node. From the jth node (internally selected by the algorithm, depending on the number of subjects), a function is generated to divide the data into the left and right child nodes. As part of the training stage, a subset of features are selected by random sampling, looking to improve the randomness in the trees of the forest. This process is repeated until arrival at a criteria point; this criteria can be established before the algorithm starts, such as the maximum of tree depth or a statistical value. The purpose of storing the statistical information in the leaf nodes is for use in future prediction.

In the testing stage, the algorithm follows a process very similar to the training stage. Initially, the subset data for testing is submitted to forest, in the root node of each tree, and it will be classified into the left or the right child node until arrival at a leaf node; this classification is based on the function learned by the training process. Each tree presents a prediction result by the statistic of the training values reserved in each leaf node, and finally, the result of the algorithm is calculated by averaging the prediction results from every tree in the forest [27].

This algorithm uses an out-of-bag (OOB) error—an unbiased estimate of the true prediction error in which each tree can be tested on the samples not used in building the tree as the forest is built. Breiman et al. demonstrated that estimating the OOB error has the same result as estimating the error using a test set of the same size as the training set [31].

An important fact of random forests is that they differ each time that they are performed; this is caused by the randomness set in the tree-building process. This randomness can be established to be always the same, obtaining specific purpose forests for certain problems [32].

RF classifier is one of the most-used machine learning algorithms, because the approach of the global interpretation involving logical relation between variables, values, and classes is very simple [33].

#### 2.2.2. K-Nearest Neighbors

K-NN is a supervised instance-based machine learning algorithm where the data given by features are clustered; this technique has been applied in different clinical imaging approaches, such as brain tissue classification in MRI data, with the aim of segmenting grey matter, white matter, and cerebrospinal fluid [34].

The nearest neighbors classification is based on the searching of the *K* prototypes nearest to the pattern to classify. The predictions are performed by associating the new examples to the examples analyzed with those that have more similarity. *K* is the most important parameter in a classification system based on K-NN algorithm; it is defined as the number of clusters of the different categories where the samples can be classified. This algorithm consists of three data sets; a training data set for the learning of the classifier, and the sets of validation and testing for proving the classifier capacity using data that is different from the training process. In the classification process, it is necessary to define a reference o test set, established as the prototype on which the nearest neighbor will be searched; then, the nearest neighbors to the test one in the training set are determined first, and its nearest neighbor is the sample that has the minimum distance to it. The measure of distance that is generally chosen to determine how close two samples are is euclidean distance, because of its generality [35].

Then, the clusters can be established according to the category distribution among these *K* nearest neighbors. The class distribution of the samples in the training set is usually irregular, meaning that clusters may have different quantities of samples between them. Finally, the algorithm determines the parameter *K*, selecting a number of clusters according to the different categories present in the data samples [36]. Then, to design the classifier, the training process is repeated with the validation set, calculating the classification percentage obtained with the examples of this set to know its specialization capacity. The last step is to evaluate the classifier capacity based on the error obtained with statistical values, classifying the examples of the test set in the *K* categories acquired with the training and validation sets [35].

#### 2.2.3. Nearest Centroid

NC is a fast and simple algorithm for classification. This classifier is a supervised method that can be considered a hybrid approach; it encompasses an instance- and a statistical-based approach. This technique has been applied in different clinical approaches, like examination feature selection techniques for protein mass spectrometry, looking for early disease diagnosis and biomarker identification [37].

The process consists of two stages—one stage for training, and one stage for testing. In the training stage, the algorithm learns with examples from a data set classifier patterns, and in the testing stage, the algorithm evaluates the classifier capacity according to the error obtained, classifying examples from a different data set than was used in the training stage. This algorithm works with any quantity of features, and its run-time complexity is proportional to this quantity.

Initially, this algorithm assumes that the target classes or categories correspond to different and individual clusters, of which it calculates their means—defined as centroids—to determine the class of a new sample point; this means that the centroid is determined as the equidistant point of the objects belonging to that cluster. Thus, for a given set of data of the same class *x*, a centroid *x* is set which, as mentioned before, is referred to the mean or the median value obtained from the data of the class *x* [33], and repeating this process, all the training set centroids are found, assigning all the examples that want to be classified in the nearest centroid—that is, whose euclidean distance is minimum. When all of the examples are assigned, it is necessary to recalculate the centroids; this process has to be repeated until a determined criteria is established. The determined criteria can be a maximum iteration number, stop having cluster changes in two successive iterations, etc. [38]. Once the final centroids are established, each unknown sample from the testing set is set to the centroid which is nearest. The classification is decided by the majority vote [39].

## 3. Materials and Methodology

In this section, a description of the public breast cancer data set used in experimentation and the methodology followed to study the behavior of the features is described.

### 3.1. Data Description

In this paper, the publicly available data set used was the Breast Cancer Digital Repository (BCDR) [21]. The BCDR data set is composed by Digital Mammography data-set number 1 (BCDR-D01) and Digital Mammography data-set number 2 (BCDR-D02). BCDR-D01 refers to a film mammography-based repository with 64 women, rendering 143 segmentations; BCDR-D02 refers to a full-field digital mammography-based repository with 164 women, rendering 455 segmentations. All subjects are in the age interval of 58 ± 11 years old, with a maximum of 89 and a minimum of 23.

BCDR is composed of 79 biopsy-proven lesions, including clinical data and image-based descriptors. All lesions are nodules or a combination of nodules with other abnormalities. BCDR is a binary class data-set due to the initial Breast Imaging Report and Database System (BI-RADS) classification of the result of the biopsy (benign vs. malignant).

A total of 37 features were extracted from each of the 598 segmentations: eight are clinical and general data, eight are intensity descriptors computed from the grey-levels of the pixels inside the lesion’s contour identified by the radiologist, 13 are texture descriptors computed from the grey-level co-occurrence matrix related to the bounding box of the lesion’s contour identified by the radiologists, and eight are shape and location descriptors of the lesion’s contour identified by the radiologists.

Any missing value is represented by the text “Not a Number” (NaN).

### 3.2. Study Design

The BCDR data-set was firstly separated in a train set comprised by 70 percent of the data-set and a test set of 30 percent of the data to do a blind validation of the results obtained, as is suggested in literature [33,40,41].

Data set analysis was realized with the training set, looking for a multivariate model with the predominant features which give the highest classification rate. The test set was used for a subsequent validation of the multivariate model obtained before, searching for a stable and accurate set of the selected features to classify the outcome (benign/malignant).

### 3.3. Data Pre-Processing

Several features were removed from the original data set because information was not relevant for this study because it was only necessary to use the automatic measures as image descriptors. Clinical and general data have to be forcibly obtained by the radiologist, and therefore, instead of reducing their work, it actually would be increased.

There were some missing values in the data, shown as Not *a* Number (NaN); these were imputed with the value calculated using the weighted average of the non-missing observations, where the weights are the proximities of the proximity matrix from the random forest with the known data.

Additionally, a Z-Norm was applied to avoid any outlier-related problems, described in Equation (10), where $x_{i}$ is the *i*^th^ value of the feature *x*, m(x) is the mean value of the feature, std(X) is the standard deviation value of the feature, and $z_{i}$ is the *i*^th^ value of the Z-Norm feature *z*. As a result, all features mean is 0 and standard deviation is 1.

(10) $$z_{i}=\frac{x_{i}-m\left(x\right)}{std(x)}$$

Later the training set and test set were manually generated, filtering the subjects with Benign outcome from the ones with Malignoutcome, and were separated using an aleatory balanced selection. The first set contained 70% of the subjects with each of the outcomes, 344 benign outcomes and 75 malignant, conforming the train set. The remaining subjects—which integrate 30% of the original database, 147 benign outcomes and 32 malign—conformed the test set.

### 3.4. Feature Selection Approach

Statistic analysis tasks were performed to generate the multivariate model: feature extraction, feature selection, model validation. All procedure were performed using R (http://www.r-project.org/).

Initially, the clinical and general data were extracted because the analysis was only focused on image descriptors, and personal data of the patient was not necessary. The features removed were: Age, Breast Density, Mammography Nodule, Mammography Calcification, Mammography Microcalcification, Mammography Axillary Adenopathy, Mammography Architectural Distortion, Mammography Stroma Distortion, Image View, and Mammography Type.

The model was constructed by a computer-assisted analysis, with a feature selection procedure by a genetic algorithm (GA) followed by an RF approach, applied to the features in the training database—reducing the size of the database features in order to obtain those that could potentially distinguish their tumor classification performance.

To apply the GA approach, the Galgo package was used. Galgo is a generic R software package that uses a genetic algorithms approach in order to optimize problems by a selection of variables (genes) and subsets (chromosomes) [33].

### 3.5. Genetic Algorithm Settings

The genetic algorithm settings were set to generate chromosomes comprised of five genes (features) selected randomly from the 27 image descriptors. These chromosomes evolved throughout 300 generations, using RF as cost function. Said parameters were chosen given the recommendation of several proposals in literature [33,42,43,44] to give statistical significance to the evolutionary process. During this process the chromosomes mutated, reproduced, and recombined, eventually yielding a highly accurate and robust model.

Fit was defined as the accuracy of the model to classify the two outcomes (Benign/Malignant), following train–test methodology. This process was set with 200 “Big Bang” objects, resulting in 200 highly accurate feature models, obtaining a five-feature model with the highest fitness. The number of times each feature was found in these models was used to determine a feature rank, which describes the potential classification capabilities of each feature.

According to this rank, forward selection and backward elimination were realized, establishing the features that were selected in the next phase of the feature selection process.

Forward selection is an outstanding method used to set up models at low computational costs. From the total of ranked features, this approach added on one feature at a time and evaluated the efficiency of the models. When last feature was added on and all the models with the different feature combinations were evaluated, the model with the features that obtained the best fitness was kept, and the rest were disregarded.

Backward elimination was used to avoid repeating information and to decrease the total number of features to be used. This process involves removing one feature in each cycle and evaluating the performance of the model, starting with the final model of the forward selection procedure, and removing from the less-frequent feature in the generated models. If this process of eliminating a feature did not affect the accuracy of the model, that feature was retracted from the final model. This process was repeated until reaching model stability [33].

The same genetic algorithm process was realized using K-NN and NC approaches as cost function, searching for stability in the features of the final model to warranty independence of the cost function.

### 3.6. Validation

To validate the results obtained the false positive/false negative rates of the model with the test set were calculated, which proved to have a similar fitness or even higher than the multivariate model with all the features included.

A statistical analysis was also conducted, obtaining the Area Under the Receiver Operating Characteristic Curve (AUC), with the purpose of knowing the specificity–sensitivity value; the Odds Ratio (OR) and its confidence intervals, with the purpose of knowing the probability of one event according to other; and the Out Of Bag (OOB) error, with the purpose of knowing the accuracy of the models obtained from each approach for comparison.

## 4. Results

To compare results, a Galgo algorithm was run with the training database using three different approaches: RF, NC, and K-NN. First, results were obtained with the RF method.

**Figure 1:**
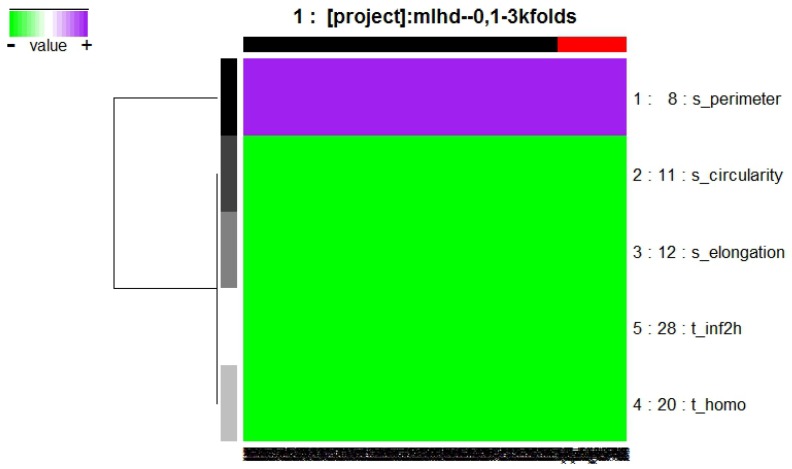
Model obtained from Galgo algorithm with Random Forest (RF) method.

The model obtained by RF approach is composed of the genes indicated in Figure 1, integrated by two texture descriptor features: t_homo and t_inf2h; and three shape and location descriptors: s_perimeter, s_elongation, and s_circularity.

The features of this model are described in Equations (2), (3), and (6)–(8).

**Figure 2:**
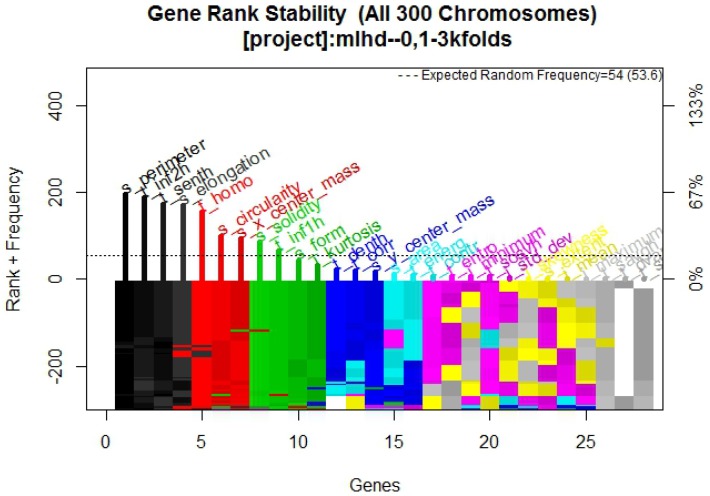
Gene rank stability graph obtained from Galgo algorithm with RF method.

In Figure 2 it is possible to observe the frequency graph for the RF approach, which indicates that the most surviving features are: s_perimeter, s_circularity, s_elongation, t_homo, and t_inf2h.

**Figure 3:**
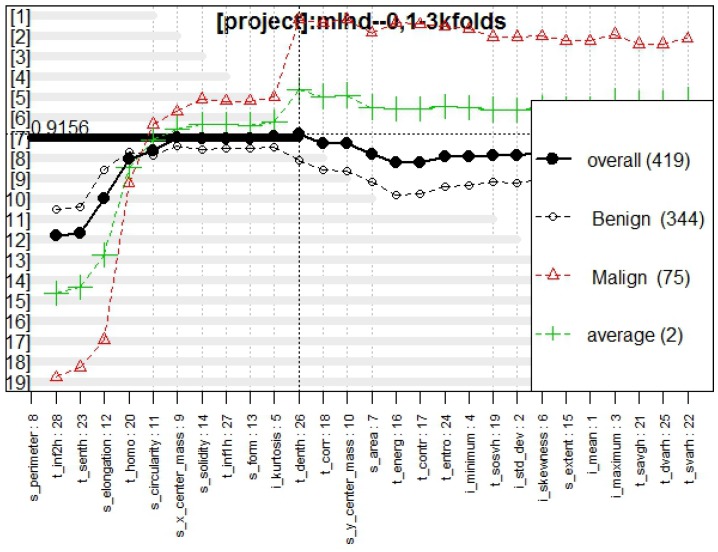
Genes behavior through Galgo algorithm with RF method.

Figure 3 presents the genes behavior, for which the best model obtained a fitness of 0.916.

The statistical values of the model obtained by this method showed an AUC of 0.938, an OOB error of 8.380%, and a total of five false negatives and ten false positives. Table 1 contains the OR values for each feature.

**Figure 4:**
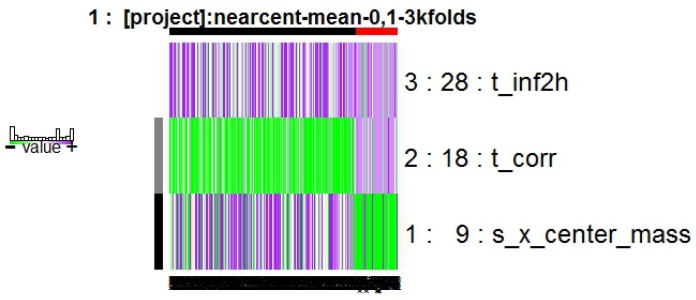
Model obtained from Galgo algorithm with Nearest Centroid (NC) method.

The model obtained by NC approach is composed of the genes indicated in Figure 4, integrated by three features: t_corr, t_inf2h (texture descriptors), and s_x_center_mass, which is a shape and location descriptor.

The features of this model are described in Equations (3), (4), and (9).

**Figure 5:**
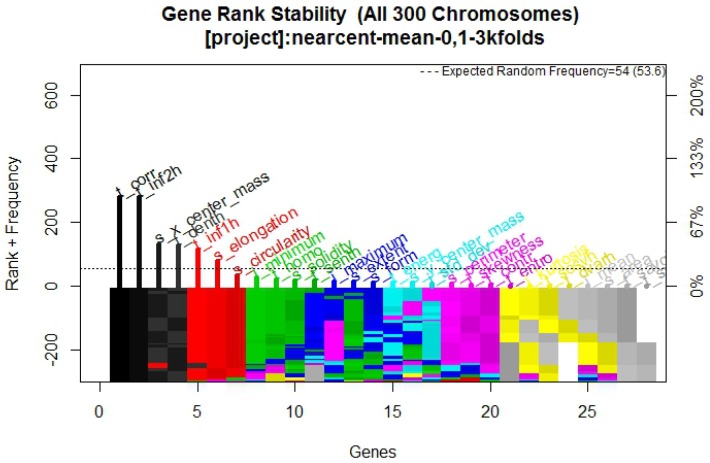
Gene rank stability graph obtained from Galgo algorithm with NC method.

The results obtained with the NC method are shown in Figure 5 displaying the frequency graph for this approach, the results of which indicate that the most-surviving features are the ones of the model obtained.

**Figure 6:**
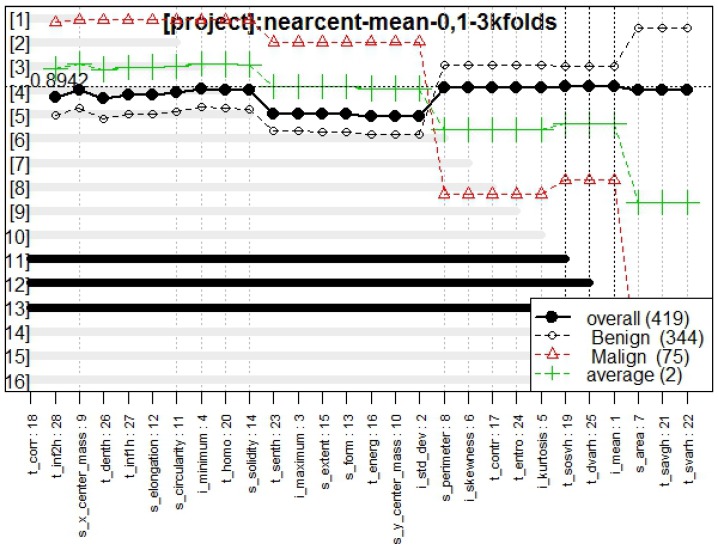
Genes behavior through Galgo algorithm with NC method.

Figure 6 presents the genes behavior, for which the best model obtained a fitness of 0.894.

The statistical values of the model obtained by this method showed an AUC of 0.962, an OOB error of 8.590%, and a total of 23 false negatives and 13 false positives. Table 2 contains the OR values for each feature.

**Figure 7:**
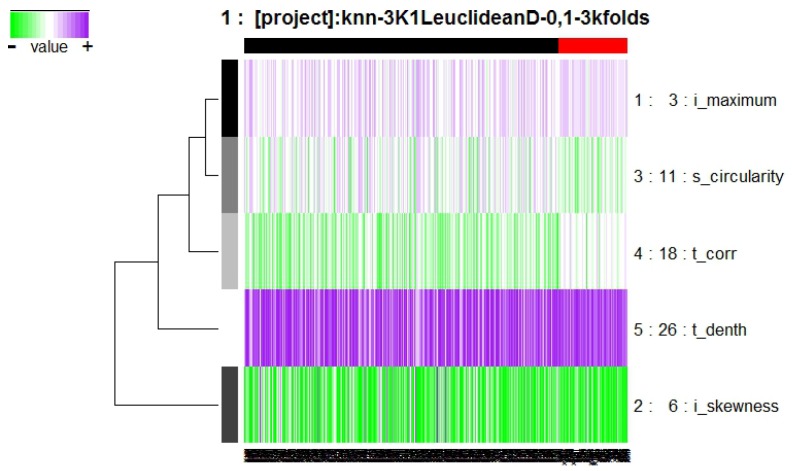
Model obtained from Galgo algorithm with K-Nearest Neighbors (K-NN) method.

Finally the results with K-NN method were obtained. This model is composed of the genes indicated in Figure 7, integrated by six different features.

The features s_circularity, t_corr, t_denth, and t_skweness are described in Equations (1), (4), (5), (7), and the feature i_maximum refers to the maximum intensity value in the region surrounded by the contour of the segmented lesion.

**Figure 8:**
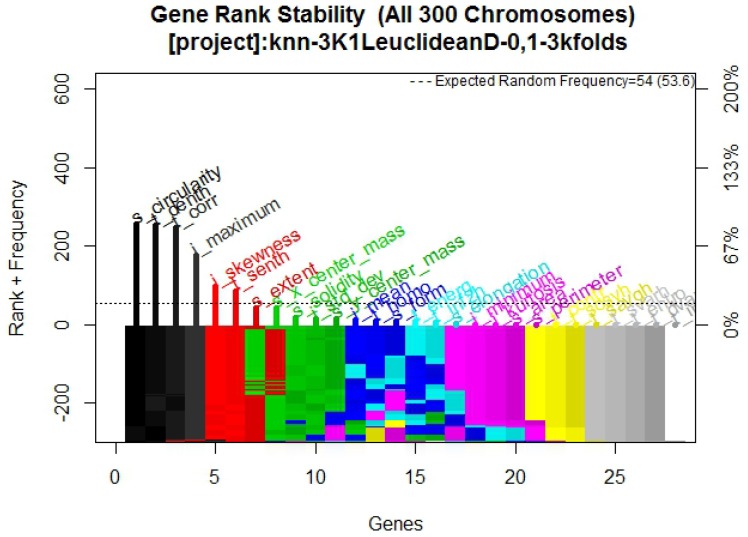
Frequency graph obtained from Galgo algorithm with K-NN method.

In Figure 8 it is possible to observe the frequency graph for this approach, the results of which indicate that the six most-surviving features are: t_corr, i_maximum, t_denth, i_skewness, and s_circularity, of which two are texture descriptor features (t_corr and t_denth), two are intensity descriptors (i_maximum and i_skewness), and one is a shape and location descriptor (s_circularity), demonstrating that these features are sufficiently predictive to correctly classify the outcomes.

**Figure 9:**
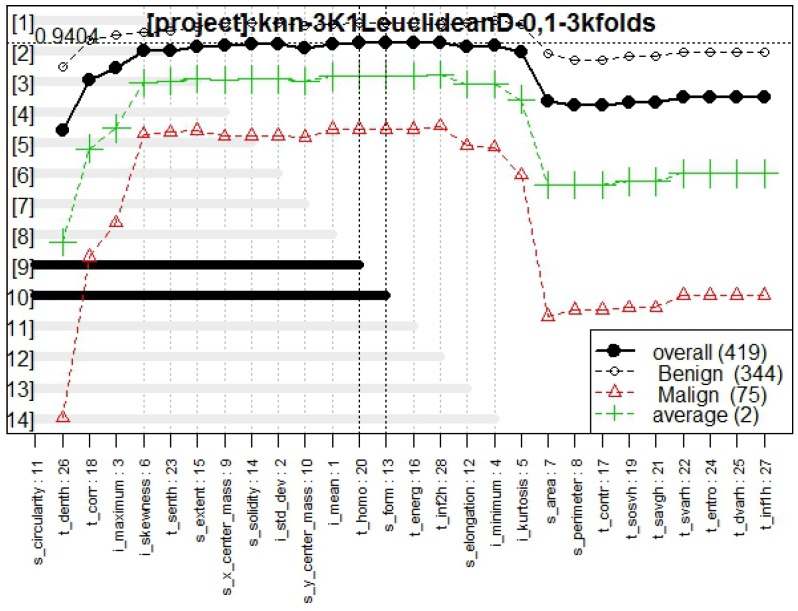
Genes behavior through Galgo algorithm with K-NN method.

Figure 9 presents the genes behavior, for which the best model obtained a fitness of 0.940.

The statistical values of the model obtained by this method showed an AUC of 0.967, an OOB error of 6.440%, and a total of 19 false negatives and 8 false positives. Table 3 contains the OR values for each feature.

Finally, Table 4 presents a comparison of the values acquired by each cost function in this study, showing that K-NN and NC had similar performance.

## 5. Conclusions and Discussion

The purpose of this research was to find a group of mammography features that efficiently describe whether a tumor is benign or malignant, in order to develop a multivariate model with CAD, using familiar machine learning techniques to confirm the features behavior. The results presented were validated with training and blind test methodology using statistical analysis, supporting the model obtained. The frequency graph in Figure 2—which presents its respective multivariate model in Figure 1 composed of two texture descriptors and three shape and location descriptors features—compared with the frequency graphs in Figure 5 and Figure 8 and their respective models, confirms that the most predictive features are those in the texture and shape and location descriptors classification from BDCR.

In Figure 3, Figure 6, and Figure 9, the behavior of models classifying tumors with all the proposed approaches are presented. From these figures, it is possible to confirm a high fitness for all of them, RF and K-NN approaches obtaining a fitness >0.9, whereas NC presented a fitness of 0.89, which is totally acceptable in the classifying task.

The statistical results (AUC = 0.936, OOB = 7.640%, false positives = 8, false negatives = 9) of the RF approach and its OR values contained in Table 1 describe a high accuracy of the multivariate model in the classifying task, showing a high sensitivity/specificity value and a low error rate. Therefore, this model can be used to predict the outcome (tumor classification) with a high confidence fitness, giving a second opinion to the radiologist and reducing their work, being that the mammography features of the multivariate model are only texture, shape, and location descriptors, making it unnecessary for the radiologist to obtain clinical and general data and intensity descriptors.

The statistical values (AUC = 0.937, OOB = 7.160%, false positives = 10, false negatives = 7) of the NC approach and its OR values contained in Table 2 with one texture descriptor feature and one shape and location feature show that the model has an accuracy very similar to the one with RF approach, keeping stability in the RF model according to the kind of features.

Table 3 includes the OR values of the K-NN approach and its statistical values are also shown (AUC = 0.967, OOB = 6.440%, false positives = 8, false negatives = 19). It is possible to observe a stability of the values in comparison with the other approaches, again affirming that these two feature classifications—texture and shape and location descriptors—are important factors to correctly classify whether a tumor is benign or malignant.

Finally, with the comparison between each approach, it is possible to validate the results obtained from RF approach, showing stability in the kind of selected features in the RF model.

The result from the genetic algorithm is a model comprising five features that can predict the outcome with a high fitness, assisting the radiologist to obtain an answer regarding the patient’s tumor, having only to extract the features of the multivariate model, significantly reducing their work and obtaining a second opinion with CAD.

The methodology demonstrated that the system has the potential to be used as a second opinion for the radiologist, or may have the practical use of triaging mammograms in developing countries where there is a deficiency of expert readers. It also has the potential to reduce the number of unnecessary biopsies, thus reducing the stress in the patient.

## Figures and Tables

**Table 1:** Odds Ratio (OR) values from the model obtained by RF method.

Features	Odds Ratio	2.5%	97.5%
s_cicularity	6.35 × 10${}^{-2}$	6.85 × 10${}^{-3}$	5.19 × 10${}^{-1}$
s_elongation	7.33 × 10${}^{1}$	6.445	8.86 × 10${}^{-2}$
s_perimeter	1.002	1.001	1.002
t_homo	1.16 × 10${}^{4}$	1.71 × 10${}^{2}$	1.32 × 10${}^{6}$
t_inf2h	8.95 × 10${}^{2}$	7.61 × 10${}^{1}$	1.35 × 10${}^{4}$

**Table 2:** Odds Ratio (OR) values from the model obtained by NC method.

Features	OR	2.5%	97.5%
t_corr	3.99 × 10${}^{4}$	5.48 × 10${}^{2}$	7.16 × 10${}^{7}$
t_inf2h	1.093	1.23 × 10${}^{-4}$	7.36 × 10${}^{2}$
s_x_center_mass	1.20 × 10${}^{-1}$	2.78 × 10${}^{-2}$	4.69 × 10${}^{-1}$

**Table 3:** OR values from the model obtained by K-NN method.

Features	OR	2.5%	97.5%
i_skewness	9.79 × 10${}^{-1}$	5.56 × 10${}^{-1}$	1.88 × 10${}^{-1}$
t_corr	2.11 × 10${}^{4}$	2.07 × 10${}^{3}$	3.64 × 10${}^{5}$
t_denth	7.61 × 10${}^{-2}$	8.25 × 10${}^{-3}$	5.28 × 10${}^{-1}$
i_maximum	4.58 × 10${}^{2}$	6.858	6.36 × 10${}^{4}$
s_cicularity	6.35 × 10${}^{-2}$	6.85 × 10${}^{-3}$	5.19 × 10${}^{-1}$

**Table 4:** Values comparison between the three cost functions: RF, K-NN, and NC.

Cost Function	False Positives	False Negatives
RF	10	5
K-NN	8	19
NC	13	23
